# Diagnostic Value of Composite and Simplified FDG-PET/CT Scores in Polymyalgia Rheumatica and the Influence of Recent Glucocorticoid Treatment—A Retrospective Diagnostic Cohort Study

**DOI:** 10.3390/diagnostics13030514

**Published:** 2023-01-31

**Authors:** Louise Schouborg Brinth, Annette Hansen, Dorte Vendelbo Jensen, Ole Rintek Madsen, Rikke Broholm, Martin Krakauer

**Affiliations:** 1 Department of Imaging and Radiology, Copenhagen University Hospital, DK-3400 Copenhagen, Denmark; 2 Department of Nuclear Medicine, Copenhagen University Hospital Herlev-Gentofte, DK-2900 Copenhagen, Denmark; 3 Department of Rheumatology, Center for Rheumatology and Spine Diseases, Rigshospitalet, Gentofte and Herlev Hospital, DK-2900 Hellerup, Denmark; 4 Department of Clinical Physiology and Nuclear Medicine, Copenhagen University Hospital Bispebjerg-Frederiksberg, DK-2400 Copenhagen, Denmark

**Keywords:** polymyalgia rheumatica, positron emission tomography, fluorodeoxyglucose, FDG, PET, glucocorticoid

## Abstract

[^18^F]Fluorodeoxyglucose positron emission tomography (FDG-PET) is increasingly used to demonstrate inflammation in specific sites typical for polymyalgia rheumatica (PMR). Scoring systems based on FDG uptake have been proposed to increase diagnostic accuracy. Methods: Retrospective inclusion of 198 consecutive patients ≥40 years of age referred for FDG-PET from the Department of Rheumatology. We assessed the degree of FDG uptake in predilection sites visually, as well as semiquantitatively, and through logistic regression analyses, we evaluated the performance of existing scoring systems as well as a new, simplified scoring system, against the final clinical diagnosis at 6 months after the FDG-PET scan. Results: We found high diagnostic accuracy for the diagnosis of PMR (range 0.74–0.91) using most of the existing scoring systems in glucocorticoid-naïve patients. A simplified scoring system including only periarticular FDG uptake in the shoulders and the ischiogluteal bursae retained high sensitivity and specificity (0.92 and 0.86, respectively). We found a detrimental effect on diagnostic accuracy in all scoring systems in patients treated with glucocorticoids within 4 weeks prior to FDG-PET. Conclusion: Most FDG-PET scoring systems perform well for the diagnosis of PMR, and there is no loss of either sensitivity or specificity in the simplest scoring systems evaluating FDG uptake in only a few selected anatomical regions. However, systemic glucocorticoid treatment up to 4 weeks prior to FDG-PET has a markedly detrimental effect on the diagnostic accuracy of all scoring systems.

## 1. Introduction

Polymyalgia rheumatica (PMR) is a rheumatic syndrome characterized by muscle aching and stiffness in the neck, shoulders, upper arms, lower back, and thighs, often accompanied by fever and fatigue.

PMR shows a predilection for the synovium of the proximal joints and bursae, and objective findings are synovitis and bursitis [1]. Substantial overlap is seen between PMR and giant cell arteritis (GCA), which is a segmental vasculitis in large and medium-sized arteries and the most prevalent vasculitis in western countries [2]. PMR is the inflammatory autoimmune rheumatic disease with the highest incidence above the age of 50 years and is largely limited to patients in this age group [3].

PMR can be diagnostically challenging, as symptoms may be diffuse and nonspecific, mimicking many other diseases, such as elderly onset rheumatoid arthritis, infections, and cancer, and an unambiguous diagnostic test is lacking. The diagnosis of PMR is still mainly clinical although supported by inflammatory markers in clinical biochemistry [1]. However, increasing evidence points to the value of an array of imaging modalities in the diagnostic workup when PMR is suspected—including ultrasound (US) of affected periarticular regions, magnetic resonance (MR), and [^18^F]fluorodeoxyglucose positron emission tomography (FDG-PET) [4]. FDG-PET is a functional imaging technique well established in oncology. It is also able to detect the accumulation of activated inflammatory cells due to their high expression of cell-surface glucose transporter proteins. Therefore, FDG-PET is increasingly used in the diagnostic and differential diagnostic workup in patients with PMR, often showing characteristic patterns of periarticular FDG uptake [5,6].

Frequently published predilection sites of increased FDG uptake in PMR are the acromioclavicular joints, shoulders, sternoclavicular joints, hips, and the symphysis pubis. Also frequently reported are the ischiogluteal, iliopectineal, trochanteric, and interspinous bursae [7]. Different scoring systems based on the assessment of FDG uptake in these sites have been proposed to optimize sensitivity and specificity for the diagnosis of PMR, some based on visual analyses and some based on quantitative analyses [7]. To our knowledge only one study by van der Geest et al. has attempted to apply previously published scoring systems in a different patient population [8], concluding that the best-performing existing scoring system was the composite score proposed by Haenckerts et al. [9], henceforth termed the Leuven score (names of the scoring systems as proposed in the publication by van der Geest et al.), and a somewhat simplified version, the Leuven/Groningen score. The originally reported sensitivity and specificity for the diagnosis of PMR with the Leuven score were 85% and 88%, respectively, performing equally well in the confirmative study (90% sensitivity and 84% specificity) [8,9]. A few groups have since proposed simplified algorithm-based scoring systems based on FDG uptake in only a few anatomical sites with only limited loss of sensitivity and specificity, the Saint-Etienne and the Heidelberg scores [10,11].

Some previous studies have indicated that recent treatment with systemic glucocorticoids may have a detrimental effect on the sensitivity of FDG-PET for the diagnosis of PMR [12,13].

The aims of the present study were:

- To evaluate the performance of the aforementioned FDG-PET scoring systems on data from our center.
- To devise an alternative, simple scoring system that is easily applicable in a daily clinical setting without sacrificing diagnostic accuracy, and to determine if simple, visual evaluations of the FDG-PET could substitute the more arduous volume-of-interest (VOI)-based analyses without loss of diagnostic accuracy.
- To assess the possible detrimental effect of recent glucocorticoid treatment on the diagnostic accuracy of FDG-PET for the diagnosis of PMR.

## 2. Materials and Methods

Patient selection: This was a retrospective study including all patients aged ≥ 40 years consecutively referred for an FDG-PET/CT scan for any indication by the Department of Rheumatology from April 2016 to April 2019. Due to the retrospective nature of the study, the requirement for informed consent was waived. A local data protection approval was obtained (#18015440). Of 258 eligible patients, 60 were excluded due to prior explicit refusal by the patients to participate in research projects, leaving 198 for further analysis.

Patient characteristics and diagnosis: PMR and/or GCA were suspected in 136 patients (69%) at the time of referral for FDG-PET/CT. Follow-up was performed by clinical experts from the referring Department of Rheumatology. Clinical and laboratory data were obtained from the electronic patient record. The final diagnosis given 6 months after the FDG-PET/CT scan was considered the reference standard for PMR. The clinical experts incorporated all available information in their diagnosis, e.g., patient records, blood biochemistry, imaging, clinical disease course, and response to pharmacotherapy. They also had access to the routine report from the FDG-PET/CT scans, but they did not have access to the diagnostic scores under investigation. Final diagnoses were categorized as PMR, GCA, PMR + GCA, Rheumatoid arthritis, other musculoskeletal inflammatory diseases, cancer, and other or no established diagnosis. Recent treatment (within 4 weeks prior to the FDG-PET scan) with systemic glucocorticoids was noted and quantified. We categorized patients treated with an intramuscular injection of 80 mg methylprednisolone as having received an equivalent daily dose of oral prednisolone 3.6 mg for 28 days.

Scan procedure: [^18^F]-FDG-PET/CT scans were performed on a Discovery GE-710 PET/CT scanner after fasting for at least 4 h, and 60 min after intravenous injection of 3.5 MBq/kg FDG. Depending on the clinical information, the scan was performed with or without intravenous contrast. PET data were reconstructed using the GE proprietary Q.Clear^®^ algorithm. Corresponding axial PET and CT reconstructions were reformatted to 3 mm nonoverlapping slices.

Image analysis: Three observers (experienced nuclear medicine physicians) evaluated the FDG-PET/CT scans blinded to patient data using MIM software version 6.9.2, MIM Software Inc., Cleveland, OH, USA. The patients were assigned to the observers based on the patient’s day of birth. The following PMR predilection sites were analyzed: acromioclavicular joints, shoulder joints, sternoclavicular joints, hip joints, greater trochanters, iliopectineal bursae, symphysis pubis entheses, ischial tuberosities, and cervical and lumbar interspinous bursae.

Semiquantitative analysis: A 3-dimensional volume of interest (VOI) was drawn manually encompassing each predilection site. For reference activity, a large VOI was drawn in the right liver lobe (avoiding liver edges and any focal and vascular FDG uptake), and a 5 (±0.1) mL VOI located inside the superior vena cava (SVC) extending caudally from the inlet of the innominate vein. For all VOIs, SUVpeak was computed as the highest possible average of voxel standardized uptake values in a 1 mL spherical kernel inside the given VOI [14]. SUVpeak was chosen in order to minimize the effect of a high SUV in a single voxel as is the case for SUVmax. The SUVpeak measures were divided by the SUVmean in the reference tissues and the resulting ratios were converted to ordinal semiquantitative scores in order to emulate the visual scores most often used in previous studies while preserving the objectivity of the scores: Score 0 (no uptake): SUVpeak ≤ SVC; Score 1 (below liver): SUVpeak > SVC and < liver; Score 2 (equal to and above liver): SUVpeak ≥ liver.

Visual analysis: In order to devise a clinically applicable intuitive tool, a visual evaluation of the same predilection sites was made using a simple 3-point scale, Score 0 (normal uptake); Score 1 (mildly increased uptake, less than liver); Score 2 (markedly increased uptake, more than liver).

Interobserver agreement: Interobserver agreement for both the semiquantitative and the visual analysis was determined through triple evaluation by the three PET readers of a small group of 10 random cases.

Evaluation of existing scoring systems: Based on the semiquantitative scores, we calculated scores corresponding to previously published scoring systems: the Leuven score [9], the Leuven/Groningen score [8], the Saint-Etienne score [10], and the Heidelberg score [11]. The latter two are based on only a few anatomical sites, whereas the Leuven and Leuven/Groningen scores are composite scores including multiple predilection sites. We were unable to calculate the Besançon score [13] as it regards the symphysis entheses as bilateral regions, whereas we did not in our analysis. An overview of the evaluated scoring systems is supplied in Supplementary Materials, Table S1.

Statistical analyses: These were performed using the IBM SPSS 25 program. Both the VOI-based analysis and the visual appraisals were represented on an ordinal scale of FDG uptake in each region. Levels of interobserver agreement were determined by Kendall’s W. The correlation between the semiquantitative and visual scores (paired regions given by mean values) and the final diagnosis (PMR/non-PMR) was determined using Kendall’s tau-b due to the nonparametric nature of the data. A Mann–Whitney U test was run to determine if there were differences in scores between patients with and without PMR (the test was only performed if visual inspection confirmed that the distributions of both semiquantitative and visual scores were similar in PMR versus non-PMR patients.)

Binary logistic regression with both forward and backward selection/elimination was used to identify the strongest association to a final diagnosis of PMR or PMR + GCA among the many predilection sites. These were subsequently included in receiver operating characteristic (ROC) analysis along with the previously published composite scoring systems. Diagnostic accuracy parameters (specificity, sensitivity, and accuracy) were calculated. Results were considered significant when *p* (two-tailed) < 0.05.

## 3. Results

### 3.1. Patients

We performed 6-month clinical follow-ups in 198 patients (120 women) of which 70 and 8 patients were diagnosed with PMR and PMR with concomitant GCA, respectively (Table 1). Eighty-eight patients (42 of the PMR patients) had been treated with systemic glucocorticoids within four weeks prior to the FDG-PET scan. Blood biochemistry and clinical criteria for diagnosing PMR, as per the EULAR provisional criteria from 2012, were recorded retrospectively [15]. However, data were only sufficient to establish a EULAR score in 27 of 198 patients, thus precluding the application of this score when establishing the clinical diagnosis. These data are shown in Supplementary Materials, Table S2.

### 3.2. InterObserver Agreement

There was a very high interobserver agreement between both ordinal measures (W = 0.949, *p* < 0.005) and visual scores (W = 0.846, *p* < 0.005) indicating that the physicians applied essentially the same standard when assessing the scans.

### 3.3. Semiquantitative and Visual Analyses

Both semiquantitative and visual scores were significantly higher in PMR patients compared to non-PMR patients in all sites except for the semiquantitative measures of the iliopectineal bursae and the cervical interspinous bursae in patients treated with glucocorticoids (Table 2). The shoulder joints and ischiogluteal bursae showed the strongest association with the diagnosis of PMR in both semiquantitative and visual scores (Table 2).

The weakest associations to the diagnosis of PMR were found between both semiquantitative and visual scores of acromioclavicular joints, cervical interspinous bursae, and semiquantitative scores of the iliopectineal bursae. Correlations were similar for semiquantitative and visual scores.

### 3.4. Simplified Visual Score

**Figure 1:**
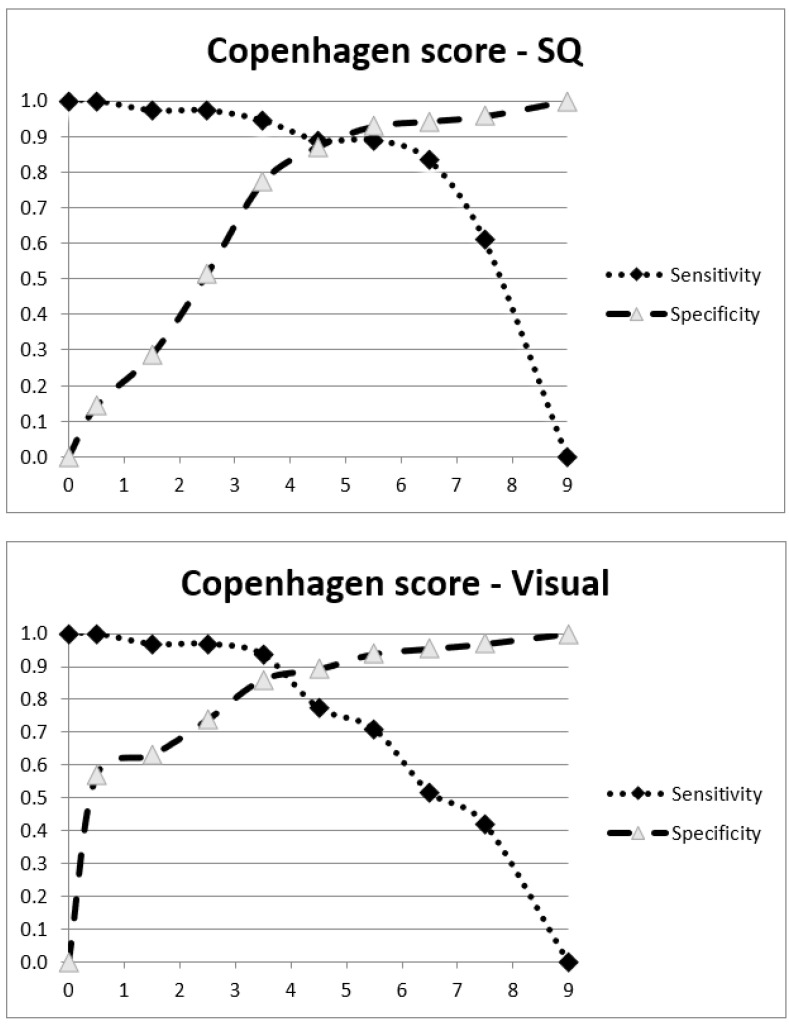
Plots of sensitivity and specificity for the semiquantitative (SQ) and visual (Visual) Copenhagen score in steroid-naïve patients.

Using binary logistic regression with both forward and backward selection/elimination, it was found that the combined summed score of the bilateral periarticular regions of the shoulder joints and the ischiogluteal bursae was the best discriminator for the final diagnosis of PMR—for both semiquantitative and visual scores (data not shown). Based on this finding, we defined a “Copenhagen score”—as the cumulated scores of only two anatomical sites, namely the shoulder joints and the ischial tuberosities (maximum score 8). The optimal cutoff was 5 for the semiquantitative Copenhagen score and 4 for the visual Copenhagen score (Figure 1).

### 3.5. Evaluation of Previously Published Scoring Systems and Comparison with the Copenhagen Score

**Figure 2:**
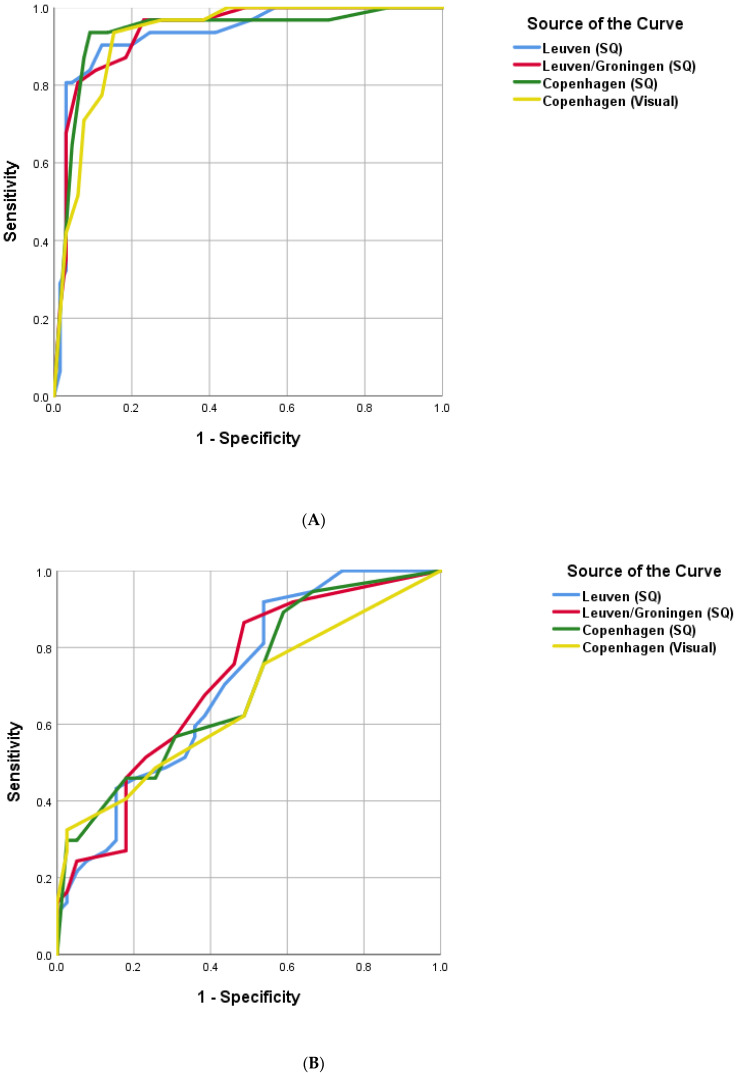
Receiver operating characteristic of four composite scoring systems. (**A**): Glucocorticoid-naïve patients; (**B**): Glucocorticoid-treated patients. SQ = semiquantitative (VOI-based) 3-point scores. Visual = Visual 3-point scores. AUC: Leuven (SQ) 0.93. Leuven/Groningen (SQ) 0.94. Copenhagen (SQ) 0.94. Copenhagen (Visual) 0.94.

We applied the aforementioned previously published scoring systems as well as our own “Copenhagen score” to the glucocorticoid-naïve patients in our dataset, as shown in the data in Figure 2A (ROC curves for the composite scores) and Table 3 (sensitivity and specificity for composite and algorithm-based scores).

### 3.6. Effect of Treatment with Glucocorticoids

**Figure 3:**
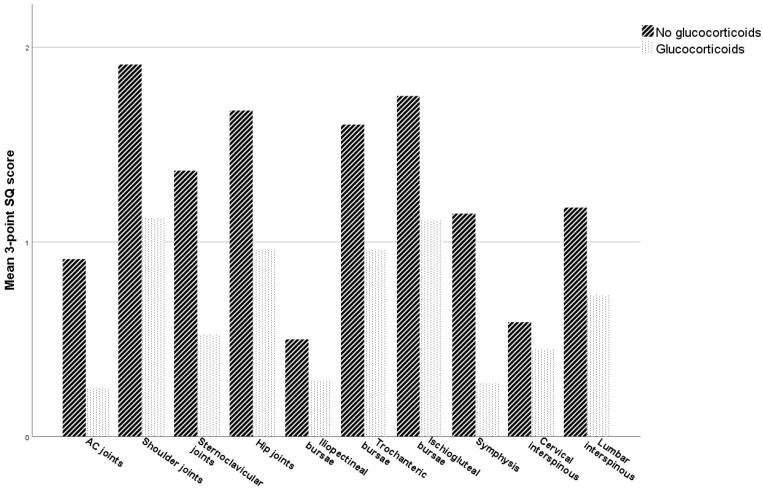
FDG uptake in predilection sites when comparing PMR patients with and without glucocorticoid treatment. Mean values of the 3-point semiquantitative (SQ) score in predilection sites for PMR patients with and without glucocorticoid treatment in the last 4 weeks before FDG-PET/CT. Paired regions are given by mean values.

We observed a marked detrimental effect on the performance of all FDG-PET scoring systems by glucocorticoid treatment within 4 weeks prior to FDG-PET (Table 3, Figure 2B). The sensitivity of all scoring systems declined markedly while specificity, not surprisingly, was relatively unaffected. FDG uptake was reduced across all predilection sites in PMR patients treated with glucocorticoids (Figure 3). We found no dose-dependence or correlation with the number of days since the latest administered dose of glucocorticoid (data not shown). However, only a few of the glucocorticoid-treated patients with PMR had paused the treatment for more than one day (7 of 42), rendering our data unsuitable for demonstrating a possible (inverse) correlation between FDG uptake and the time since the last dose.

## 4. Discussion

In this retrospective study of FDG-PET as a tool in the diagnosis of PMR, we were able to confirm the good performance of selected previously published scoring systems in glucocorticoid-naïve patients. The Leuven, Leuven/Groningen, and Heidelberg scores all performed well with high diagnostic accuracy, whereas the Saint-Etienne score was hampered by only moderate specificity. These findings are in good agreement with a recently published evaluation of the scores [8]. Additionally, we were able to devise a simplified scoring system based on the visual appraisal of FDG uptake in only two anatomical regions without sacrificing diagnostic accuracy. Importantly, we demonstrated a marked detrimental effect of glucocorticoid treatment prior to the FDG-PET scan on the diagnostic accuracy of all scoring systems.

In contrast to the composite scoring systems [8,9,13], which are based on cumulative scores of FDG uptake in multiple articular and periarticular regions, the simplified scoring systems are based on FDG uptake in only a few selected regions that carry the most information for discriminating PMR from non-PMR [10,11]. FDG uptake in a single region is neither sufficiently specific nor sensitive, but the assessment of FDG uptake in three, or even two, regions still carries a high diagnostic accuracy. The selected regions vary somewhat between the proposed scoring systems but most often include the periarticular shoulders, interspinous bursae, ischiogluteal bursae, and trochanteric bursae. In the current study, we found that the evaluation of FDG uptake in only two regions, namely the periarticular shoulders and the ischiogluteal bursae (the “Copenhagen score”), retained high diagnostic accuracy. Thus, the more elaborate scoring systems seem to provide little, if any, added value. Interestingly, we found only modest associations between the diagnosis of PMR and both semiquantitative and visual scores of interspinous bursae, which is in contrast to previous findings [8,9,10,11]. The reason for this is unclear.

If scoring systems are to be used in a daily clinical setting, simplicity is paramount. In addition to our data indicating that FDG uptake in only two anatomical sites exhibits high diagnostic accuracy, we also found no clear benefit of quantitatively comparing FDG uptake to the uptake in reference tissues such as the liver. A simple 3-point visual scale performed equally well regarding sensitivity. However, our data do indicate that using a semiquantitative approach, referencing the FDG uptake in the liver and blood pool, might be more robust in terms of improved inter-rater reproducibility. Semiquantitative measures also allow a wider margin for the cutoff value (see Figure 1 where the sensitivity and specificity of the semiquantitative score show a more pronounced plateau around the cutoff value than with the visual score).

An important finding in this study was that glucocorticoid treatment, even up to 4 weeks prior to FDG-PET, had a markedly detrimental effect on the performance of all of the scoring systems. This has also been indicated in previous studies [12,13]. We found no dose-dependence or inverse correlation to the number of glucocorticoid-free days prior to the FDG-PET. However, as the study was not designed to determine dose- or time-dependence, this may represent a statistical type 2 error, but it may also reflect a true long-term effect of even brief treatment with systemic glucocorticoids. While a referral bias may exist in this retrospective study, possibly implying a more severe clinical disease or a more complex clinical presentation in the patients where treatment with glucocorticoids was deemed necessary, this bias would, on the contrary, be expected to decrease the detrimental effect on the scoring systems.

A study by Nielsen et al. evaluated the influence of glucocorticoid treatment on the diagnostic accuracy of FDG-PET in patients with GCA [16]. They demonstrated a “window of opportunity” of three days after the initiation of glucocorticoids where FDG-PET maintained a high diagnostic value, whereas treatment longer than 10 days prior to FDG-PET attenuated the diagnostic value of FDG-PET dramatically. We need further studies evaluating the effect of glucocorticoid treatment on FDG uptake in the predilection sites of PMR by performing FDG-PET scans before and at various time points after the initiation of glucocorticoid treatment.

The present study is one of few studies including a large number of unselected patients with a wide range of final diagnoses, which is a good reflection of the daily clinical challenge when distinguishing patients with PMR from patients with other rheumatic diseases. A weakness is the retrospective design of the study and the fact that the findings on the original FDG-PET report could have influenced the final clinical diagnosis, thus introducing an incorporation bias. We sought to minimize this by using the six-month-long clinical follow-up period until the final diagnosis was established, allowing the clinical experts to assess the overall disease course, including the response to pharmacotherapy. Despite these efforts, we cannot exclude a bias in the final clinical diagnosis, and this is a weakness inherent to the retrospective study design. Consequently, further validation of the simplified scoring systems in other cohorts with varying disease prevalence is desirable, preferentially in prospective, blinded study designs.

## 5. Conclusions

In glucocorticoid-naïve patients, simple FDG-PET scoring systems involving only a few anatomical regions such as the one developed in the current study are useful for identifying patients with PMR with high diagnostic accuracy in a mixed cohort of rheumatological patients. Treatment with glucocorticoids prior to FDG-PET is of major concern regarding the diagnostic sensitivity of PMR in FDG-PET scoring systems. It is possible that a more holistic, gestalt-like approach to the evaluation of the scans, contrary to the more rigid scoring systems, is more robust to this effect, but this remains to be shown. We encourage prospective studies on the exact clinical value of the scoring systems and of the effects of prior treatment with glucocorticoids.

## Figures and Tables

**Table 1:** Patient characteristics stratified by clinical diagnosis after 6 months.

*Variable*		All (*n* = 198)	PMR or PMR + GCA (*n* = 78)	Non-PMR (*n* = 120)
**Age (years)**		68 ± 11 (range 42–92)	71 ± 9 (range 46–88)	66 ± 12 (range 42–92)
Sex (female/male)		120 (61%)/78 (39%)	45 (58%)/33 (42%)	75 (63%)/45 (37%)
Diagnosis				
	PMR	70 (35%)	70 (90%)	-
	GCA	14 (7%)	-	14 (12%)
	PMR + GCA	8 (4%)	8 (10%)	-
	Rheumatoid arthritis	24 (12%)	-	24 (20%)
	Other inflammatory diseases	29 (15%)	-	29 (24%)
	Cancer	8 (4%)	-	8 (7%)
	Other/no objective disease	45 (23%)	-	45 (38%)
Glucocorticoid ≤ 4 weeks before FDG-PET		88 (44%)	42 (54%)	46 (38%)

Data are presented as mean ± SD or *n* (%).

**Table 2:** (**a**) Mean values of semiquantitative and visual scores in glucocorticoid-naïve patients by all referral diagnoses (*n* = 198). (**b**) Mean values of semiquantitative and visual scores in glucocorticoid-naïve patients suspected of PMR at referral (*n* = 119).

	Semiquantitative Measures				Visual Measures			
	PMR	Non-PMR	*p*-Value	Kendall’s τ_b_	PMR	Non-PMR	*p*-Value	Kendall’s τ_b_
(**a**)								
Acromioclavicular joints	0.90	0.47	0.003	0.265	0.92	0.40	0.001	0.311
Shoulder joints	1.89	0.88	<0.001	0.590	1.57	0.51	<0.001	0.553
Sternoclavicular joints	1.35	0.38	<0.001	0.534	1.19	0.24	<0.001	0.562
Hip joints	1.68	0.63	<0.001	0.536	1.23	0.6	<0.001	0.559
Iliopectineal bursae	0.50	0.23	0.122	0.145	0.75	0.12	<0.001	0.506
Trochanteric bursae	1.60	0.83	<0.001	0.470	1.18	0.46	<0.001	0.453
Ischiogluteal bursae	1.67	0.45	<0.001	0.611	1.47	0.25	<0.001	0.657
Pubic joint	1.08	0.33	<0.001	0.389	0.72	0.11	<0.001	0.435
Cervical interspinous bursae	0.56	0.24	0.016	0.227	0.42	0.11	0.004	0.276
Lumbar interspinous bursae	1.11	0.57	0.001	0.306	0.89	0.19	<0.001	0.428
(**b**)								
Acromioclavicular joints	0.93	0.54	0.034	0.246	0.94	0.35	0.002	0.359
Shoulder joints	1.94	1.09	<0.001	0.674	1.61	0.75	<0.001	0.502
Sternoclavicular joints	1.33	0.52	<0.001	0.466	1.20	0.31	<0.001	0.501
Hip joints	1.73	0.69	<0.001	0.587	1.28	0.25	<0.001	0.580
Iliopectineal bursae	0.52	0.29	0.245	0.145	0.78	0.25	0.004	0.370
Trochanteric bursae	1.65	0.92	<0.001	0.477	1.22	0.54	0.001	0.442
Ischiogluteal bursae	1.70	0.41	<0.001	0.667	1.50	0.26	<0.001	0.637
Pubic joint	1.09	0.44	0.008	0.327	0.74	0.22	0.010	0.319
Cervical interspinous bursae	0.57	0.33	0.163	0.172	0.43	0.19	0.110	0.200
Lumbar interspinous bursae	1.11	0.48	0.003	0.362	0.91	0.19	<0.001	0.429

Paired joints/bursae are given by mean values of the paired foci. Comparison of scores between patients with and without PMR determined by Mann–Whitney U test (*p*-value) and correlations between scores and the diagnosis of PMR by Kendall’s τ_b_. PMR = polymyalgia rheumatica.

**Table 3:** Diagnostic performance (PMR or PMR + GCA versus other diagnoses) of different scoring systems in glucocorticoid-naïve patients versus patients treated with glucocorticoids. The originally reported data are stated in the last three columns.

	GC-Naïve Sensitivity % (CI)	GC-Naïve Specificity % (CI)	GC-Naïve Accuracy % (CI)	GC-Treated Sensitivity % (CI)	GC-Treated Specificity % (CI)	GC-Treated Accuracy % (CI)	Reported Sensitivity (%)	Reported Specificity (%)	Cutoff Score (≥)
Leuven SQ [9]	81 (63–93)	95 (87–99)	91 (83–96)	24 (12–41)	93 (80–98)	60 (49–71)	85 †	88 †	16
Leuven/Groningen SQ [8]	84 (66–95)	89 (79–96)	88 (79–94)	35 (20–53)	83 (68–93)	60 (49–71)	90	84	8
Saint-Etienne SQ [10]	84 (67–95)	69 (57–80)	74 (64–83)	53 (36–69)	65 (49–79)	59 (48–70)	79 ‡	80 ‡	-
Heidelberg SQ [11]	86 (71–95)	91 (82–97)	90 (82–95)	36 (22–52)	77 (62–88)	57 (46–68)	91	92	-
Copenhagen SQ	89 (74–97)	87 (77–94)	88 (80–93)	43 (28–59)	72 (57–84)	58 (47–68)	-	-	5
Copenhagen Vis	92 (78–98)	86 (75–93)	88 (80–93)	38 (24–54)	80 (65–90)	60 (49–70)	-	-	4

GC = glucocorticoid. SQ = semiquantitative VOI-based 3-point score. Vis = visual 3-point score. † = nonattenuation-corrected PET. ‡ = glucocorticoid-treated patients included in the cohort.

## Data Availability

The data presented in this study are not publicly available in full due to the sensitive personal information contained in the data files.

## Supplementary Materials

The following supporting information can be downloaded at: https://www.mdpi.com/article/10.3390/diagnostics13030514/s1, Table S1: Previously published scoring systems; Table S2: Supplementary patient characteristics stratified by clinical diagnosis after 6 months.
