# Supplementary material for: Diagnostic Value of Composite and Simplified FDG-PET/CT Scores in Polymyalgia Rheumatica and the Influence of Recent Glucocorticoid Treatment—A Retrospective Diagnostic Cohort Study
Source: Diagnostics (Basel). 2023 Jan 31;13(3):514. doi: 10.3390/diagnostics13030514 (PMC9914179; doi:10.3390/diagnostics13030514)
Supplement: Supplementary file 1 [file diagnostics-13-00514-s001.zip › diagnostics-2118334-supplementary.pdf]

Supplementary Table S1

Previously published Scoring systems

Scoring system	Regions evaluated	Scores	Max score	Cut-off criterion	Remarks	Reported Sensitivity	Reported Specificity
Leuven [9]	Cervical spinous processes, lumbar spinous processes, left and right sternoclavicular joint, left and right ischial tuberosity, left and right greater trochanter, left and right hip, and left and right shoulder	0: no FDG uptake<br>1: moderate FDG uptake, less than liver uptake<br>2: intense FDG uptake, equal or more than liver uptake	24	≥16	Non-attenuation corrected PET	85%	88%
Leuven/Groningen [8]	Sternoclavicular joints, hips, ischial tuberosities and lumbar interspinous bursa	0: no uptake<br>1: uptake lower than liver<br>2: uptake equal to liver<br>3: uptake higher than liver.	14 (cumulated score in sites with score ≥2)	≥8		90 %	84 %
Besançon [13]	Two shoulders, two acromioclavicular and two sternoclavicular joints, two greater trochanter, two hips, two ischial tuberosities, two iliopectineal bursae, two symphysis pubis enthesis and the most inflammatory interspinous bursa.	0: indicating no uptake (same as bone)<br>1: slight uptake<br>2: moderate uptake (same as liver)<br>3: uptake higher than the liver	17 (number of sites with a score ≥2)	≥3		74 %	79 %
Saint-Etienne [10]	Two shoulders, two acromioclavicular joints (AC joint), and two sternoclavicular joints (SC joint), the most intense interspinous bursa, two hips, two trochanteric bursas (TB), two ischial bursas (IB), two iliopectineal bursas (IPB), and two symphysis pubis enthesis (SPE).	0: no uptake<br>1: uptake lower than the liver<br>2: moderate uptake, same as that of the liver<br>3: higher uptake than the liver	n/a	Interspinous bursa score ≥ 2 and/or trochanteric bursa score ≥2	Glucocorticoid treated patients included	79 %	80 %
Heidelberg [11]	Two periarticular shoulder regions, two glenohumeral joints, two acromioclavicular joints, two sternoclavicular joints, two periarticular hip regions, two hip joints, two trochanteric regions, regions adjacent to the two ischial tuberosities, two knee joints, two posteromedial knee regions and most PET avid interspinous bursa	0: no uptake<br>1: lower than normal liver uptake<br>2: similar to normal liver uptake<br>3: higher than normal liver uptake	n/a	Ischial tuberosity score ≥2 and (periarticular shoulder ≥2 or interspinous bursa score ≥2)		91 %	92 %

Supplementary Table S2

Supplementary patient characteristics stratified by clinical diagnosis after 6 months

	PMR or PMR+GCA (N=78)	Non-PMR (N=120)
C-reactive protein [mg/L]<br>median (range)	20 (2-381)	11 (1-194)
Anti-CCP<br>(Pos / Neg / Not measured)	2 / 58 / 18	13 / 69 / 38
Rheuma-Factor<br>(Pos / Neg / Not measured)	2 / 34 / 42	15 / 40 / 65
Morning Stiffness > 45 minutes<br>(Yes / No / Not recorded)	39 / 13 / 26	30 / 34 / 56
Hip Pain or limited range of motion<br>(Yes / No / Not recorded)	27 / 14 / 37	12 / 38 / 70
Abscense of other joint involvement<br>(Yes / No / Not recorded)	22 / 38 / 18	24 / 73 / 23
EULAR score ≥ 4<br>Yes / No / Insufficient data to calculate	11 / 3 / 64	6 / 7 / 107
